# “Has It Really Changed, or Does It Just Look Different?”: The State's Administration of Domestic and Family Violence Service Delivery in Australia as Contiguous Colonial Violence

**DOI:** 10.1177/10778012251410217

**Published:** 2026-02-03

**Authors:** Emma Buxton-Namisnyk

**Affiliations:** 1 University of Oxford, Oxford, UK; 2 UNSW Law and Justice, Law and Justice Building, UNSW Sydney, NSW, Australia

**Keywords:** domestic and family violence, state violence, domestic violence specialist services, violence against First Nations women

## Abstract

This article examines how Australia's approach to domestic and family violence (DFV) social service delivery impacts First Nations community-controlled DFV services and theorizes how administrative techniques used by the state in this context can be considered a form of state violence. Analyzing data from a multi-stage qualitative study involving 22 interviews alongside analysis of 98 DFV-related intimate partner homicides involving First Nations women, this article argues that the state's approach to funding and managing DFV service delivery sustains a contiguous colonial logic that threatens and undermines the strength and resilience of community-controlled service delivery and, as a consequence, harms First Nations women.

## Introduction

Domestic and family violence (DFV) is a serious social issue in Australia. It is estimated that one in four Australian women has experienced emotional abuse (and one in six physical violence) from a current or former intimate partner since the age of 15 (Australian Institute of Health and Welfare, 2025a), one in three Australian men has admitted to using intimate partner violence as an adult (O’Donnell et al., 2025), and, on average, one woman is killed by an intimate partner every 11 days (Australian Institute of Health and Welfare, 2025b). First Nations women in Australia account for about 3% of Australia's female population (Australian Bureau of Statistics, 2022) and are overrepresented as victims of DFV. First Nations women are 32 times more likely to be hospitalized due to DFV than non-Indigenous women and 11 times more likely than non-Indigenous women to be killed in a fatal assault (Australian Human Rights Commission, 2020, p. 44). First Nations women, scholars and advocates have long criticized the nation's silence and inaction around violence against First Nations women, noting the ongoing dominance of white, middle-class women's interests within the Australian DFV movement: a silence and apathy inextricable from Australia's colonial context (see, e.g., Ingram, 2016; Loney-Howes et al., 2024; McGuire, 2018).

First Nations people have long argued that to prevent and respond to DFV, victim-survivors must have access to culturally safe and community-led services (Aboriginal and Torres Strait Islander Women's Task Force, 1999; Aboriginal Legal Services NSW/ACT, 2022; Cunneen, 2010; Langton et al., 2020; Royal Commission into Family Violence, 2016; The Special Taskforce on Domestic and Family Violence in Queensland, 2015, p. 123–124: 33; The Senate, 2024). However, despite First Nations women repeatedly, and over many decades, reinforcing the need for greater investment in self-determined solutions to DFV (Aboriginal and Torres Strait Islander Women's Task Force, 1999; Cunneen, 2010; Langton et al., 2020; The Senate, 2024), service responses developed with the non-Indigenous population in mind, especially the criminal legal system and “mainstream” specialist organizations, continue to dominate Australia's DFV response landscape (Loney-Howes et al., 2024).

This article examines Australia's specialist service response to DFV, drawing on findings from an iterative, qualitative, two-stage study of First Nations women's service contact histories in fatal intimate partner DFV cases. The first stage involved the examination of DFV death review (“DFVDR”) and coronial files of First Nations women who were killed by, or killed, an intimate partner following a history of DFV victimization (*N* = 98) across most Australian states/territories (New South Wales, Victoria, Queensland, South Australia, Tasmania, and the Northern Territory, excluding Western Australia and the Australian Capital Territory). The second stage involved interviews and yarning (discussed further in Method) with 22 First Nations DFV specialist workers ('specialist workers”), DFV survivors, and Elders. Findings from the first stage of the study revealed that First Nations women had low levels of contact with DFV specialist services prior to homicide, particularly when compared with their high levels of contact with police (considered elsewhere, Buxton-Namisnyk, 2022). In exploring this finding, participants in the second stage of the study described challenges with the administration, including the funding and regulation, of First Nations community-controlled organizations delivering DFV services. Many participants also described these economic and regulatory processes as colonial state violence, thus founding this article's theoretical focus.

This article accordingly highlights the ways in which Australia's administrative approach to DFV service delivery, developed with non-Indigenous service delivery and non-Indigenous people in mind, is racialized and antagonistic to the diversity represented by First Nations organizations and people. Drawing explicitly on observations made by study participants, this article also observes that given Australia's colonial context, seemingly neutral and largely invisible regulatory responses associated with the administration of DFV service delivery should be understood as a form of state violence that sustains a contiguous, colonial logic and harms First Nations women.

## Context and Terminology

Australia was invaded by the British in 1788. As Wolfe (1999, p. 2) has described, in Australia, processes of invasion and colonization were undergirded by the logic of elimination and extinction; the settlers “came to stay.” Colonization dispossessed First Nations people from their ancestral lands, which were then exploited economically by the colonizers for their own benefit and that of the British crown (Lange et al., 2006). Colonization also encompassed cultural (Nayton, 2011; Renes, 2011) and biological genocide via the removal and attempted forced assimilation of First Nations children (Tatz and Higgins, 2016). Australia was “settled” without a treaty or constructive arrangement being negotiated with First Nations peoples, and non-Indigenous Australians and governments continue to occupy unceded First Nations lands (Jonas, 2002). First Nations resistance to the practices and processes of colonization has continued since 1788, including against the criminalization and deaths in custody of First Nations people (see, e.g., McGuire, 2020; Whittaker, 2021), contemporary child removal practices (see, e.g., Davis, 2019; Newton et al., 2024), and conceptualizations of, and responses to, DFV that have displaced community-led solutions (see, e.g., Cripps, 2023; Loney-Howes et al., 2024).

Australia's colonial context is central to this article's analysis and its argument about state violence. As many First Nations and non-Indigenous scholars have argued, Australia is not a post-colonial state, but a state defined by ongoing occupation and unceded First Nations sovereignty (see, e.g., Heiss, 2003).

This article adopts the terminology of “the state” throughout. In Australia, this must be understood within a colonial context. As Sullivan (2018, p. 201) describes

at its most abstract [the state] is an assemblage of coercive practices tending always to reinforce existing relations of power founded in control of the economy. These practices are instituted by the state's various organs—the judiciary, the police and defence forces, education and the parliament as a whole.

Sullivan's definition not only supports the theorization of state power as nebulous and diffuse but also provides a useful framework to illuminate the myriad ways in which state power, founded in colonial domination and perpetuated through contemporary policies, practices, and approaches to service delivery, impacts First Nations peoples.

## Specialist Domestic and Family Violence Service Funding

In Australia, alongside the criminal legal system, specialist DFV services, including shelters/refuges and counseling/support services, are a central pillar of the state's response to DFV (Murray and Powell, 2011). Unlike the criminal legal system, which is funded and delivered by the government, DFV specialist service delivery is mostly outsourced and provided by non-government organizations (“NGOs”) or charities through grants of public funding.

Public sector outsourcing of social services has a long history in Australia but has been particularly prominent since the 1980s with the implementation of New Public Management (“NPM”) (also known as Neoliberal Public Management) (O’Flynn, 2007). NPM essentially sought to reframe public sector relationships to be based on economic market relationships; outsourcing policy, implementation, and delivery functions via contracts, and subjecting contracting processes to administrative technologies such as competitive tendering and deregulation (O’Flynn, 2007; Sullivan, 2018). In the area of social service delivery, NPM created new economic markets where they did not previously exist (Dean, 1999), and today this has translated into social service delivery frameworks in which external providers enter into service-delivery contracts with government agencies, subject to reporting requirements against stipulated performance indicators, metrics, and outcomes (Figgis and Griffith, 1997).

When it comes to DFV services, due to Australia's federalist structure, the Commonwealth (federal) government and states and territories are responsible for administering different aspects of outsourcing and procurement (Langton et al., 2020, p. 75). It has been estimated that there are in excess of 1,000 DFV services in Australia that receive funding under Commonwealth and state/territory funding schemes (Cortis et al, 2018).

## First Nations Service Delivery Funding

In addition to state and Commonwealth funding specifically for DFV service delivery, organizations may also seek Commonwealth funding to deliver services, including DFV-related services, to First Nations peoples. Relevant schemes include the *Indigenous Advancement Strategy* (“IAS”), which administers funding across a range of portfolios, including Commonwealth funding related to First Nations women's safety.

As Sanders describes, during the *Aboriginal and Torres Strait Islander Commission* (“ATSIC”)-era, mainstream services operated alongside Indigenous services as a form of “supplementary service delivery” (see Howard-Wagner et al., 2018, p. 22–23). However, after ATSIC (a statutory First Nations representative body) was dismantled by the Australian government in 2005, “standardized government programs” often replaced the “Indigenous specific programs” the government inherited from the agency (Howard-Wagner et al., 2018, p. 22). As Howard-Wagner (2018, p. 218) observes, under this “new mainstreaming” approach

Aboriginal organisations were no longer to be subsidised by the state. They were no longer to be given special treatment. This placed many existing urban Aboriginal organisations in funding competition with secular and religious non-government organisations. They were also subject to a whole new set of regulatory arrangements that dictated the way this newly defined social service sector did business with government (see also Moran et al., 2016).

While Commonwealth funding reforms such as the IAS were designed to create solutions to this crippling administrative burden (Liberal Party of Australia, 2013, p. 43), the IAS has been criticized for reducing and mainstreaming funding of service-delivery of Indigenous programs (Page, 2018, p. 189–190). The IAS was initially introduced quickly, resulting in community-controlled organizations being required to competitively tender against one another and compete with mainstream services for funding (Page, 2018, p. 186, 189). When it was initially implemented, the majority of federal funding for First Nations service delivery was allocated to non-Indigenous organizations, and only 21% went to First Nations organizations (Henderson, 2015; Howard-Wagner, 2018, p. 219). As Page (2018, p. 186) describes (see also, Sanders, 2002; Sullivan, 2011; Strakosch, 2015; Sanders, 2018), the IAS continues

the neoliberal governance mechanisms and audit technologies used by the Commonwealth in Indigenous affairs policy practice from the mid-2000s, following the dissolution of [ATSIC] as a pillar in an ‘Indigenous order of Australian government’ and a ‘shield’ from major changes in Australian public administration.

While today, following the Auditor General's damning review of the IAS (ANAO, 2017), its funding guidelines have been reviewed, including to enhance their accessibility and “prioritize” service-delivery by organizations that involve First Nations people, non-Indigenous organizations can still seek, and be granted, contracts for service delivery (National Indigenous Australians Agency, 2021). It is also not yet clear to what extent recent shifts in governmental policy frameworks, for instance, to the community-led governance partnership model embodied in the *Closing the Gap Partnership Agreement* (Commonwealth of Australia, 2019), may improve service delivery opportunities for community-controlled DFV organizations. While a genuine partnership approach, should it translate into improved administrative management, has the potential to overcome many issues identified in this article, a recent 2024 Productivity Commission review highlights that to date governments have underdelivered against the partnership aspects of the *Closing the Gap Partnership Agreement*—describing that this underperformance “raises questions about whether governments have fully grasped the scale of change required to their systems, operations and ways of working to deliver the unprecedented shift they have committed to” (Productivity Commission, 2024, p. 3). A 2025 Aboriginal and Torres Strait Islander-led independent review of *Closing the Gap* further describes that due to the government's lack of systemic action to unsettle racist and government-led ways of working, there had been a “lack of transformational change” among services and systems (Jumbunna, 2025, p. 5). That report also reinforced that “funding reform is required to provide for sustainable, long-term security to the community-controlled sector” (Jumbunna, 2025, p. 9).

## Challenges Facing First Nations Community-Controlled Organizations Delivering Domestic and Family Violence-Related Services

Research conducted over the past two decades has identified that First Nations community-controlled organizations delivering DFV services face numerous ongoing challenges.

### Underfunding of Services

Underfunding of services is a key concern that has consistently been identified (see, e.g., *Aboriginal and Torres Strait Islander Women's Task Force on Violence*, 1999, p. 123; *Victorian Indigenous Family Violence Task Force* 2003, p. 57, 80, 81). As was described in Victoria's *Royal Commission into Family Violence* (2016, p. 50)

[l]ong standing under-investment, combined with increasing demand for services due to population growth and increased levels of reporting, means that Aboriginal organisations and communities are struggling to deliver their own solutions.

That report (2016, p. 50) further noted that

in examining government funding data it is apparent that there is a pattern of relatively small allocations of funding spread widely across a number of organisations. This occurs both in short-term and ongoing funding streams. This may reflect responsiveness on a geographic basis, the emergence of particular projects over time, a high degree of fragmentation or potentially all three.

### Funding Practices

The processes by which community-controlled organizations delivering DFV services are required to apply for, and secure, funding have also been subject to criticism related to their bureaucratic inaccessibility. Concerns were raised in submissions to the *Aboriginal and Torres Strait Islander Women's Task Force on Violence* (1999, Annexure 2, Submission 10) that service funding guidelines are inaccessible, adopt bureaucratic language, and that “[c]ommunity people often don’t know, [and] aren’t informed about available funding” (Annexure 2, Submission 31 and 32). The *Victorian Indigenous Family Violence Task Force* (2003, p. 118) similarly identified that workers may lack resources to write funding submissions or tenders, and, over a decade later, in 2016, Victoria's *Royal Commission into Family Violence* (2016, p. 31) described that community organizations have to repeatedly ask for funds and re-apply year after year, without any certainty that their programs will be continued, even if they are very successful (see also, even more recently, Aboriginal Family Legal Service Southern Queensland, 2020; Djirra, 2020; The Senate, 2024, p. 160–162). In Victoria's *Royal Commission into Family Violence* (2016, p. 30–31), funding practices were described as being “disdainful to clients” and “a disincentive for people in need of assistance to use services because of lack of continuity.”

There is also evidence that the IAS’s implementation negatively impacted community-controlled DFV services. In Langton et al.'s site-based research, the researchers described that community-controlled DFV organization, Djirra, had experienced significant funding cuts when the IAS was introduced in the 2013/2014 funding cycle and, as a result, Djirra now worked on a (more destabilizing) 1–3-year funding cycle (Langton et al., 2020, p. 76).

It has also been identified that where funding is provided, allocations may also be insufficient or inadequate for organizations to flexibly deliver the full range of DFV-related services the community needs (Aboriginal Family Legal Service Southern Queensland, 2020). For instance, submissions to the *Aboriginal and Torres Strait Islander Women's Task Force on Violence* (1999: Annexure 2, Submissions 20, 25, 33, and 34) described that community safe houses (refuges)—while being increasingly used by community members—were under-resourced and, accordingly, were not always open. Two decades after that Task Force reported, these concerns continue to be raised (see, for instance, NATSIWA, 2020, p. 5–6). It has also been observed that there may be a lack of funding for adequate staffing or staff training, including for specialist roles (Aboriginal and Torres Strait Islander Women's Task Force on Violence, 1999: Annexure 2, Submissions 31 and 32) and to support women's leadership (Aboriginal Family Legal Service Southern Queensland, 2020). Inadequate funding may also limit opportunities for capacity-building, which may have a cyclical impact on a service's ability to deliver its services and secure funding (Aboriginal and Torres Strait Islander Women's Task Force, 1999: Annexure 2, Submissions 30 and 31; Victorian Indigenous Family Violence Task Force, 2003, p. 116, 118; Djirra, 2020, p. 9).

### Program Evaluations

Limited or inadequate program evaluation has also been identified as a factor that may impact the amount of funding that First Nations community-controlled organizations receive and diminish their funding competitiveness. In the mid-2000s, Memmott et al. (2006, p. 94) described that few Indigenous DFV programs were evaluated, and in 2014 the Victorian Auditor-General (2014, p. x) reaffirmed that—in general terms—there still remained “significant scope” to improve monitoring, evaluation, and reporting of First Nations service delivery strategy and program outcomes. As Mugford and Nelson (1996, p. 2) describe, many projects run on small budgets, and the cost of evaluation would outweigh the cost of running the program. They observe (p. 2) that “much more investment in evaluation is needed, but not at the expense of precious operational dollars.” Evaluation can also be undermined by a lack of data or limited access to data (Memmott et al., 2006, p. 95). Further, it has been observed that evaluation approaches and measures may also need to look different for First Nations communities (Williams, 2018) and may be an inappropriate driver for change given structural inequality and political imbalances between First Nations people and the state (Dillon, 2020).

While these DFV-related service delivery challenges facing community-controlled organizations have been routinely identified in inquiries and studies over the past two decades, to date, these have not been the subject of in-depth qualitative research. Drawing directly on the observations of First Nations service workers, Elders, and DFV survivors, this article progresses this analysis.

## Method

The data in this article originated from a two-stage, qualitative research study conducted from 2017–2019. The study was designed with First Nations stakeholders through scoping discussions in 2016. The study initially involved examination of all 98 cases of DFV-related intimate partner homicide involving a First Nations woman as a homicide victim (*N* = 68) or perpetrator (*N* = 30) that occurred between 2006 and 2016 in New South Wales, Queensland, the Northern Territory, Victoria, South Australia, and Tasmania. In all cases studied, First Nations women had been victims of DFV prior to the homicide. Case identification was undertaken, and original files were sought from DFVDRs in each of the jurisdictions studied, as well as coroners in jurisdictions without DFVDR bodies. DFVDR or coronial files were accessed in person (Victoria, South Australia, Tasmania), de-identified case narratives were provided (Northern Territory, New South Wales) or a mix of narratives and original case files were used (Queensland). This case analysis process focused on women's service contact histories preceding homicide.

The second stage of the research involved interviews and yarning (Dean, 2010; Powers, 2004) with 22 First Nations specialist workers, DFV survivors and Elders, as well as some observation. Group yarning is an Indigenous research method that aims to create respectful sharing and telling spaces where people can meet to discuss and resolve issues (Dean, 2010; Powers, 2004). This part of the research also involved spending time at a First Nations community-controlled organization offering DFV services. This included observation, as well as speaking with workers delivering DFV services and services in related areas (Figure 1).

**Figure 1.**
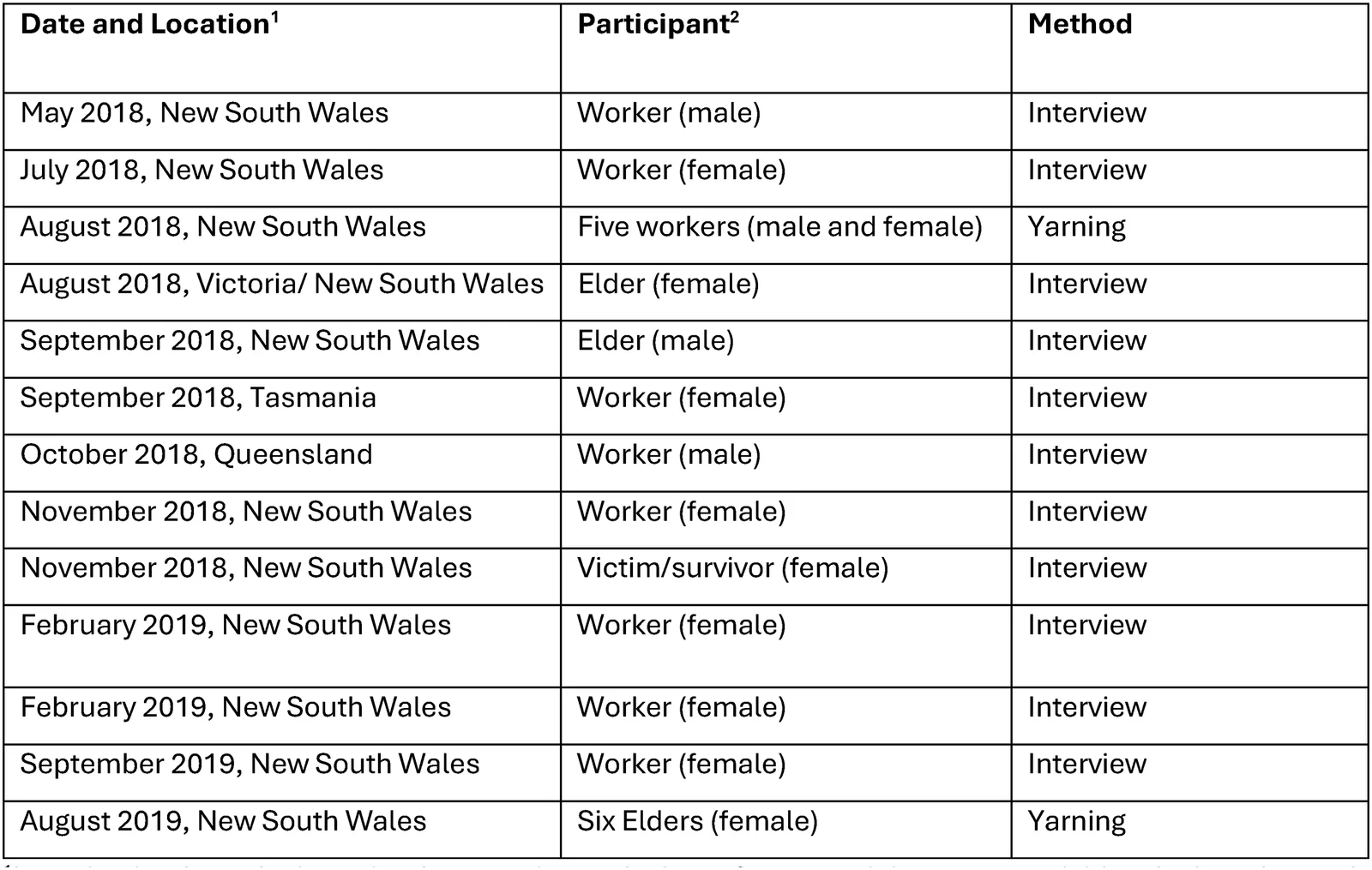
Interview/yarning participants (*N* = 22).

*Many participants were workers as well as Elders and/or victims/survivors. **Some interviews involved participants visiting from other jurisdictions. Participants had often worked or lived in multiple jurisdictions and regional/remote/rural areas.

In the second stage of the research, participants were asked questions about the state's response to DFV against First Nations women, the criminal legal system, the availability, accessibility and quality of DFV services, successful community approaches, the adequacy of current approaches to participation and self-determination in this area, and potential ways forward. These questions were exploratory in nature. Importantly, the research process was iterative (Mills et al., 2010); case findings structured interview questions, and the cases were revisited following interviews and yarning. The data were coded in NVivo using a mixture of deductive and inductive coding. Study findings were discussed, disseminated, and shared with participants both at the conclusion of data analysis and again at the end of the study.

## Findings

### Case Study Findings

Across the homicide cases studied, it was a key finding that First Nations women had very limited contact with DFV specialist services prior to episodes of fatal violence. In comparison to the high levels of contact women had with police in relation to DFV (*N* = 88, 90% of the women who were killed by, or killed, an abusive partner), a finding considered elsewhere (Buxton-Namisnyk, 2022), comparatively few women had contact with mainstream/settler-controlled or First Nations community-controlled DFV services. In total, just over a quarter of First Nations women whose cases were examined in the study had contact with a First Nations community-controlled or mainstream service (*N* = 26, 27%). Of these women, five had contact with a community-controlled service/s only in relation to DFV, 16 had contact with a mainstream service/s or refuge/s only, and five had contact with both a community-controlled service/s and a mainstream service/s. Overall, First Nations women in the cases had much more contact with mainstream DFV services (*N* = 21) than with First Nations community-controlled DFV services (*N* = 10), noting that service contact figures were low across both service types. It was not always clear why First Nations women had contact with mainstream instead of community-controlled services (or vice versa), but it was suspected that women's service contact patterns may have related to issues of service availability, service acceptability, and service accessibility (Figure 2).

**Figure 2.**
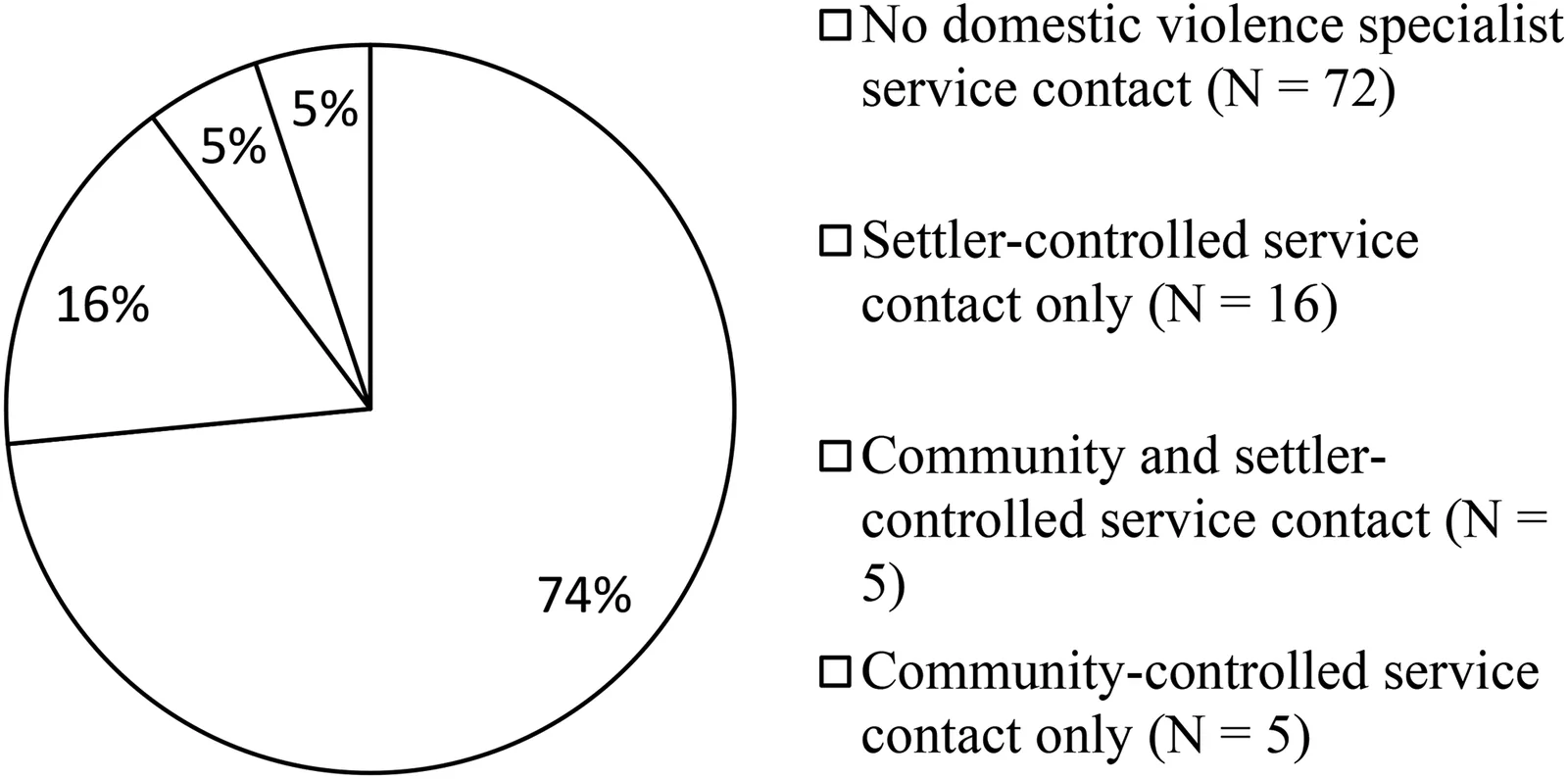
Domestic violence specialist service contact (*N* = 98).

### Interview, Yarning, and Observation Findings

During interviews and yarning, when exploring these findings, participants made a range of observations which almost exclusively focused on service-level governance issues related to the state's administration, regulation, and management of DFV-related service delivery.

#### Mainstreaming

Several participants described the ways in which First Nations community-controlled DFV services have been affected by a broader move in Australia towards the mainstreaming of social service delivery to First Nations people. As one specialist worker described in an interview,

there's the move to the mainstream in Aboriginal Affairs more generally. So, things like tendering out of services tends to favour the larger providers at the expense of specialist services, particularly for specialist Aboriginal services.

Other specialist workers specifically discussed the effects these policy approaches have had on First Nations community-controlled organizations delivering DFV-related services, describing that community organizations may be disadvantaged compared to large, mainstream NGO/charitable providers when it comes to competing for funding. In contrast to large-scale NGO/charitable providers, community-controlled organizations are often small and focused on local service needs. As one specialist worker observed in an interview,

[our] service is quite small. So, in our services, do you know who does the funding? Me…. But the thing is, like, you know, you know we aren't one of those big organisations that have those people that come in and do report write, not report writing, for funding, like … all that stuff. And when you're actually trying to provide a, you know, provide a service, what's your priorities? …[G]etting money in the door is important, but actually servicing your community is important.

These findings suggested that government funding structures and expectations particularly disadvantaged community-controlled organizations, diverting workers’ energy away from service delivery and toward (an expected and necessary) engagement with bureaucratic funding requirements. This appeared likely to either reduce a service's capacity to deliver services or, alternatively, limit a service's ability to seek funding. Both outcomes were evidently harmful to First Nations organizations seeking to deliver DFV services and First Nations women needing to access those services.

#### Assimilating Community-Controlled Organizations Through Westernized Approaches

Several participants described that government funding processes and grants models do not match holistic approaches to service delivery by First Nations DFV organizations, nor do they reflect the service needs of First Nations peoples. One specialist worker described in a group yarn that “when you get services that are funded, they’re still funded under a Western view … the workers then have to assimilate into the government's way of working.” In an interview, another specialist worker reflected on the way her organization had an application partially funded under the IAS, with the important, holistic, healing work the organization was offering being disregarded by the *National Indigenous Australians Agency* (“NIAA”—the Commonwealth agency that administers IAS funding). She described that her organization applied for funding

to do more holistic [work with families following DFV], and, but, you know, [NIAA] came back and said, “Well no, we're not gunna fund those bits of culture,” and the working with Elders and you know, what we really wanted in terms of self-determination so we could actually change … so they chose this bit and said “We’ll fund you that”.

Several workers similarly identified that for community-controlled organizations the funding awarded would be inadequate for the services an organization was required to deliver. As one worker described

there's … issues with the size and capacity of Aboriginal services as well, because … even though they’re small and, and have specialist focus—the burden placed on them within their communities is quite large. So, for an Aboriginal medical service for instance, their role is primarily providing healthcare to not just necessarily Aboriginal people in a community, but they’re providing community healthcare. But, … they do much more than that. The … burden placed on them is much more than that. So, they do the drug and alcohol services, the counselling, the DFV work, and that stretches their resources, and it also means that, that their capacity is stretched trying to meet that need.

This perspective was also reinforced during site visits to a community-controlled organization delivering DFV services during the study, where a worker described that the organization “ran on the smell of an oil rag” and that there was nowhere near the amount of funding for the burden of need in the community. Considerable service brokerage (the allocation of additional funds to contribute to central service delivery) was required at that service to fulfill demand. Another worker similarly observed in an interview that, in her jurisdiction, there was a pressing need for DFV services, but funding often did not extend to those programs. She described that

[t]hey have a lot of services that focus on specific stuff, but not specifically family violence. I don't know that they do a lot of work on that. [They should be doing more and] I think that their workers, they would agree with that too. Each worker would do it in a different way. Try to address it, but they don't have specific funding for it. They don't have that specific role in doing it. This is my impression.

These observations suggested that the state's imposition of siloed approaches to service delivery limited First Nations organizations’ ability to deliver the necessary programs to communities. This approach appeared antithetical to the need for holistic service delivery and also highlighted how responsive, reactive, and creative organizations would have to be to cope with the limitations built into government funding models and grants.

#### Community Competition and Managing Dissent

Several Elders in a group yarn described that in their community, another language group had control of community-controlled services in the town. Those participants observed that a lack of services for different language groups within particular areas could make community-controlled services inaccessible and would either force women to access mainstream services or stop them from accessing services altogether. A specialist worker, in an interview, similarly described the way the government's funding processes may fracture communities, stating

it doesn't matter what service you need, this is what's available. Then the community fights over that money and if a community's fractured, you know because this service got the money and we didn't, so we're not going to use that service. I'm going to tell everybody that's in our little loop not to use that service. They're just dividing and conquering in our own people again. We don't make the decisions. That's the difference. You know?

One specialist worker also described in an interview how state funding agreements could be used to control dissent by First Nations peoples and organizations, stating that “[it's important] … to be able to have an opinion which is not that of … the direct funding sources.”

These findings highlighted the hidden ways in which funding processes may produce tension within communities, and the ways in which they could serve to limit First Nations peoples’ ability to speak out against the state. Within Australia's colonial context, this appeared as a practice that was, on a generous reading, dismissive of the reality of First Nations’ diversity and, at worst, suggestive of a strategic decision to immobilize First Nations resistance.

#### Capacity-Building, Demonstrating Success and Trust in Communities

Several workers reflected on the ways in which funding regimes may not support capacity-building to equip community-controlled organizations to compete in social service funding environments. One specialist worker described that

I think non-Aboriginal people unfortunately [don’t] … give us the skills to equip ourselves or not give us the funding or the resources to be able to … develop ourselves, they're happy to do that. And so they can always turn, like, a blind-eye to a service going down because of that. “[The community organisation] didn't do the reports, they didn't do this, they didn't do that”. Well, what did [the government] do? What did they put in place to give them opportunities to develop those kind of … things. I mean, I was looking at governance-type training … and you know, I've seen some for like $200 a person, $205 a person…. So, it's like, you know, well, have more of this funded through government, have more of these staff going out there and doing that, because there are lot of people on the ground that can do these jobs, and can do that, and can run organisations. But it's just those things that, they get absorbed in doing the service provision … and not the account, the other part of it…

Participants also described the ways in which under-evaluation of organizations’ DFV programs (including failures to build evaluation capabilities into funding grants, or to support evaluation of community-driven programs), and a preference for innovation, could impact the funding competitiveness of community-controlled services. One specialist worker described in an interview that the government's preference for innovation impacted her service, observing that with governance and funding approaches

it's like, let's do something innovative. Everything has to be innovative. But, why? because there's things that do work, and just haven't been resourced properly to be able to do them. So, it's like we're looking at funding now, and it was like, oh, it has to be innovative. It can't be an enhancement of what you [already do]. Well, sorry, we do some stuff really well, but we just don't do enough of it because we can't. We don't have the capacity … and there's other services that do that across the board, so, stop worrying about everything being innovative. Let's just start worrying about what's worked, what does work, what the communities think works.

Another specialist worker similarly described that he had seen community-based sport-focused anti-violence programs be very successful in reducing DFV in First Nations communities. He described, however, that those programs would struggle to demonstrate their success and, under current funding arrangements, they would need to do this to be competitive. Describing the programs, he observed that

they’re so organic and community-driven. The last, the last one, I rang up the guy who's run it, and he's like, just some Aboriginal footy coach … I said, ‘You got any data?’, and you know, he goes, ‘What's this data you’re talking about brother?’ You know? I said to him, ‘Well, anything you’ve written up?’, and he said, ‘Well you can come down here and write about it if you wanna.’ He said, ‘We just doing this, you know?’ … the worst possible outcome is that what we say is that there's no evidence that these programs work…. And you’re actually going, ‘actually, you’ve never even bloody evaluated it.’ You know? How would you know? Um, all you’ve done is some international literature trawl, in which none of this kind of stuff, cause it's not in the literature yet…

While the specialist worker discussed above criticized the state's preference for innovation, another worker observed that governments may, in contrast, resist community innovation where it may pose a risk of failure. He described how, in his experience, governments may prefer to import DFV programs from overseas rather than support and trust communities to develop their own programs. He also described that the very features of overseas programs that made them successful—such as community consultation and structural support—would never be replicated when that program was brought into the Australian context. He stated that

[u]nfortunately we also import a lot of Indigenous theories from overseas … and just dump it in in Australia and go, ‘This’ll work!’…. There's also a reticence to try new things in government, because like, there's a fear of failure and we know that in order to come up with good solutions, you have to fail many times…. Because you have such risk-averse government, governments … they’re unwilling for things to fail. So that they, they want proof that something's gonna work before you’ve even tried it. So, what do we do? We import things, because we can point to evidence to say, ‘it worked in Canada. It worked in the reserves in the United States’…. ‘It worked in New Zealand’…. And then we bring it in, and it's like, no, it's a dismal failure. Why was that? Because, like it had ten times the amount of money … over there it was done through … consultation!…. It was managed through like, the governance systems…

That worker further described how, in his view, this reflects the government’s lack of trust in communities. He suggested that this distrust is imbued with racist public attitudes stating

government doesn’t trust communities because of that fear of failure that they’d be on the front page of the Telegraph [conservative newspaper] and, it's on the front page of the Telegraph today for fuck's sake!

Another specialist worker similarly reflected on the ways this lack of trust may manifest in reduced service funding for community-controlled DFV services, describing that

[a]t a national policy level, it's fundamentally an issue of trust and trust and control. But I think it's probably to do more with trust of Aboriginal communities, Aboriginal people and Aboriginal organisations being able to do their job better, or in a different way, and do it well … as opposed to the standard, traditional kind of, institutional approaches. So … and that's gonna be a bit harder to change, even though we can kinda clearly show that most of our Indigenous organisations and community-controlled organisations have done really well over the last 20–30 years, compared to other small businesses in Australia, etc. And you only ever hear about things falling over from governance. You know, you hear about it all the time. But mostly they’re small things, and mostly they’re governance issues, not practice issues. And there's a whole range of issues around that as well. And, you know, we did a kind of a look at a community-controlled health organisation here versus the state health around this issue of trust, and I’ve put together a table which, you know, contrasted, ‘Did we employ Dr. Death?’, ‘No’. Um, ‘Did a Tahitian Prince run off with $15 million of our money?’, ‘No’. ‘Did we employ nurses that weren’t actually registered as nurses?’, ‘No’. And, ‘Are we in debt $80 million, like the local district?’, ‘No’ … but where's the trust? The trust in them, and not in us?…. Australian systems have failed in Aboriginal affairs. Pretty much. And, so it's time to think about, you know, real community-grounded approaches. Not just listening to people and saying, ‘That's a good idea’, and patting them on the head and going away and doing what you like. But going, how could we possibly work with this mob to get better methodology, funding, and a good evaluation so we can show it, you know?

Some participants described, however, that notwithstanding evidence of success or positive evaluations, governments may nonetheless make funding decisions to defund or dismantle community-controlled DFV programs. One Elder observed in an interview that

the anecdotal evidence might be that [the program's] fantastic [but whether its evaluated or not] it doesn't make any difference…. It has to be deliberate, because you couldn't logically look at these things and say, “Well, that program's working really well. Let's cut it.” Who's the idiot making those decisions?

These findings again demonstrated how participants saw government funding approaches and expectations as being specifically enmeshed with racialized, colonial attitudes toward First Nations peoples. Challenges that organizational workers experienced maneuvering for funding in these complex funding environments strongly suggested a lack of trust, a disregard for the diversity and individuality of First Nations peoples and communities, and, ultimately, disrespect and paternalism.

#### Exercising Agency

While the governance practices outlined so far in this article may have detrimental effects on First Nations community-controlled organizations delivering DFV services, this has not stopped organizations from exercising agency and self-determination in this space and demonstrating considerable achievement, strength and resilience (Howard-Wagner, 2018).

This was the case for the organization visited during fieldwork, which had innovatively secured mainstream funding to deliver a DFV support program to non-Indigenous, as well as First Nations, clients. As a result, the uptake of First Nations women's participation in that mainstream program was very high. That service, however, also experienced demand that consistently exceeded its funding allocation, with significant brokerage being required to prevent the service from having to close its books to new clients.

Reflecting on the success of community-controlled organizations more generally, one specialist worker in an interview described the challenges associated with exercising agency and seeking community control in service-delivery. He observed

when you talk about community-controlled organisations who are trying to self-determine, and it's a real structured self-determination; we have to fight for every inch of that progress, or every dollar that we get, you know? So, a health service delivery organisation shouldn’t be needing to go to Canberra and Brisbane to talk to Health Departments and Ministers every couple of weeks, you know? [But they do.]

This not only demonstrated the strength and resilience of community-controlled organizations but reinforced the way state processes and expectations created barriers to that success.

#### Administrative Governance as State Violence

Several specialist workers expressed concerns about the practice of mainstream services being granted significant funds to work with First Nations clients, including under schemes such as IAS. One specialist worker described, in a group yarn, that there are “not enough funded services that are run by our mob” and another worker added that “none of [the services delivering programs] are [community-controlled], you know? They're all prescribed services, they’re not run or owned by Aboriginal people.”

Participants also described that governments awarding funding to non-Indigenous organizations to work with First Nations people may result in services that are not fit for purpose, including due to a lack of cultural safety. As one service worker observed, even though mainstream services may get the funding, “the funding they get, they got to tick boxes for it, so, at the end of the day, the services don't meet the needs of [First Nations] people.”

Some participants also specifically described that mainstream services may lack the experience or expertise to work with First Nations clients. As one specialist worker described

in the city and the regional areas, like, there's more services available. So it's a different issue where those services really need to be looking at. If its culturally-safe and really working on getting Aboriginal communities into their service.

Another specialist worker similarly described that

a lot of the services always ask why [are] communities not accessing our services? Instead of thinking, “What can we be doing to engage the Aboriginal community?” So I think there needs to be a more like a shift in services. Like not looking at Aboriginal communities saying, “why aren't they accessing” rather than, “what can we be doing?”

One specialist worker also specifically related funding governance and procurement favouring mainstream services to the state favouring non-Indigenous over Indigenous-led solutions and to the state seeking to maintain control over First Nations peoples. She described that the

Aboriginal person is always the assistant…. Let the Aboriginal people or the community organisations lead these programs in communities. Why does it always have to be a non-Aboriginal leading the way? … [even] if an Aboriginal person says something the last phrase is always by the non-Aboriginal person.

Several participants specifically connected the administrative regulation of community-controlled DFV services to ongoing forces of colonialism and state violence. As one specialist worker observed in that conversation

I think what underpins all of this, we still, even before we start talk about services, lack of services, there's still so much underpinning racism. We haven't shifted. Colonisation happened 200 something years ago but … when you're on the ground and you're seeing what's happening in our communities, it hasn't shifted.

Other workers built on these observations, describing

P2: I'd love to see a paper that's written on that false concept that we're talking about here. You can hear it. It's in everything we're saying. It's around exactly how it looked way back then, still looks the same. [The government] must look like [its] doing something, but not to make it look like, to make the community look good. Because if they looked, you know, we need [First Nations people] to continue to sabotage themselves, because then we can keep saying it's an Aboriginal problem then. [Its like this poem I wrote]…. It says like, you know, to sum it up very quickly, it talks about, “Has it really changed or does it just look different?” So, it talks about our struggle and it's that constantly, they just make it look different.

P1: Yeah, all the time. They're shifting it.

P2: It's the same shit over and over again … with a different name and a different face.

P3: We're still forced into assimilation. They just do it … saying they don't … now, but we still have to assimilate.

P4: As Aboriginal people, we don't get to be Aboriginal people. There's still assimilation and comes in legislation policy, like [Participant 3 is] saying. Not enough funded services that are run by our mob. All of that stuff…

These findings reinforce that many participants saw the processes and expectations around DFV service delivery as a calculated, and contiguous, colonial, exercise of state power. Rather than amounting to an inert and politically meaningless inconvenience, funding and regulatory approaches associated with DFV service administration were specifically identified as a deliberate strategy by the state to procure forced assimilation of First Nations community organisations (and by proxy, peoples).

## Discussion

Study participants clearly and persuasively described a range of ways in which economic and regulatory practices associated with the outsourcing of DFV service delivery had a detrimental and harmful impact on First Nations community-controlled organizations, and as a result, First Nations women experiencing DFV. Approaches such as service sector mainstreaming, failures to evaluate programs or build capacity, a lack of trust and control of dissent were all identified as practices that undermined self-determined, community-controlled DFV solutions and damaged the capacity of community organizations to deliver services to First Nations women. These practices, while administered by state bureaucrats working behind closed doors, were explicitly identified as a calculated violent strategy undergirded by the same sustained colonial logic that has impacted First Nations peoples in Australia since 1788. This understanding of the harms associated with outsourced social service delivery as a form of violence aligns with observations that have been made by scholars such as Strakosch (2018), who has argued that economic Indigenous policy maneuvers “domesticate” First Nations people and seek to reinforce the legitimacy of the state. They also reinforce that racism is a “golden thread” which links historical and contemporary regulatory practices of the state (Jumbunna, 2025, p. 108). Findings in this article highlight the ways in which this domesticating logic and racism are sustained through the DFV social service sector. That these harmful economic and regulatory processes and practices undermine responses to such a common and serious form of gender-based violence compounds their violent effect.

If the state is understood as an assemblage of coercive practices that reinforce existing power relations founded in control of the economy (Sullivan, 2018), the state's imposition of funding regimes and outsourcing methods that result in the underfunding and the undermining of First Nations community-controlled organizations delivering DFV-related services should be specifically recognized as a form of state violence (and indeed a form of state violence that disproportionately impacts and harms First Nations women). As findings within this article reinforce, this is a clandestine form of violence that may look “value neutral” through seemingly objective and bureaucratized regimes and processes, but it is a form of violence that harms the capabilities of First Nations organizations, undermines their diverse, community-focused service offerings, and threatens their strength and resilience. In this way, such economic and regulatory practices not only attempt to control and assimilate First Nations organizations and peoples but, in doing so, can be seen to preserve and reinforce state power consistent with the ideology of Australia's (incomplete) settler colonial project (Wolfe, 1999).

Despite the many harms of social service delivery outlined in this article, it is vitally important to reiterate and acknowledge the strength and resilience of First Nations community-controlled organizations that manage to not only successfully navigate these harmful, bureaucratic, and disdainful funding environments but also consistently self-determine and provide culturally safe support to First Nations women who experience DFV. As Antoinette Gentile, the then Acting CEO of community-controlled DFV organization Djirra, described of that organization in Australia's recent inquiry into *Missing and Murdered First Nations Women and Children*, “Aboriginal women matter to us, and we must matter to governments” (The Senate, 2024, p. 66).
